# A cohort study on the A8G6 neutralizing antibody intranasal spray for preventing SARS-CoV-2 infection in healthcare workers

**DOI:** 10.1016/j.gendis.2025.101617

**Published:** 2025-04-01

**Authors:** Shu Zhang, Zhigang Chu, Siyuan Yang, Qiuling Shi, Qinghua Zhao, Dawei He, Hongmei Xu, Dan Zhang, Zhu Yang, Ailong Huang

**Affiliations:** a The First Affiliated Hospital of Chongqing Medical University, Chongqing 400013, China; b Chongqing Medical University, Chongqing 400013, China; c The Second Affiliated Hospital of Chongqing Medical University, Chongqing 400013, China; d Children's Hospital of Chongqing Medical University, Chongqing 400013, China

SARS-CoV-2 has evolved dramatically since the COVID-19 pandemic in Dec 2019, acquiring mutations that enhance transmissibility, infectivity, and immune evasion.^1^ Unlike other variants targeting lungs, Omicron altered entry pathways and promoted replication in the upper respiratory tract, resulting in more asymptomatic cases. This facilitated its spread and posed challenges to infection prevention.^2^ Healthcare workers face a higher risk of being infected and should seek to protect themselves from virus transmission while providing medical care for patients.^3^ Passive antibody therapy based on neutralizing antibodies has been found to protect susceptible populations with moderate to severe immunodeficiency or vaccine contraindications. Antibodies MY-586 and MY-558, obtained from recovered patients of SARS-CoV-2 infection, have shown strong neutralizing efficacy against various mutant strains of SARS-CoV-2.^4^ Preclinical toxicity studies in mice, monkeys, and healthy volunteers revealed that A8G6, the intranasal spray formulated with MY-558 and MY-586 at a mass ratio of 4:1, had high tolerance and no serious side effects. It has been confirmed to have potent efficacy in preventing SARS-CoV-2 infection in close contact with COVID-19 patients.^5^ Our study aimed to evaluate the efficacy and safety of A8G6 in safeguarding healthcare workers in field hospitals.

This study involved a prospective cohort of field hospital workers from Nov 16 to Dec 14, 2022. Participants, including doctors, nurses, technicians, and admin staff, were trained on A8G6 intranasal spray use. The inclusion criteria comprised negative nucleic acid tests, an understanding of the trial, effective contraception, and the ability to comply. The exclusion criteria included cognitive impairments, allergies, previous coronavirus infections, and participation in other trials. Study procedures involved electronic consent, baseline questionnaires, and daily follow-ups. The primary outcomes included nucleic acid conversion rates, while the secondary outcomes focused on the use of the intranasal spray and symptoms in the treatment group. Follow-up data were collected via WeChat applets over 14 days, including symptoms, medication usage, and intranasal spray details. The study process is illustrated in Figure 1. This study has obtained approval from the Ethics Committee of the Second Affiliated Hospital of Chongqing Medical University (approval number: 2022127-1) and was filed with the Ethics Committee of the First Affiliated Hospital of Chongqing Medical University. All participants have been fully informed and signed the informed consent forms.

**Figure 1:**
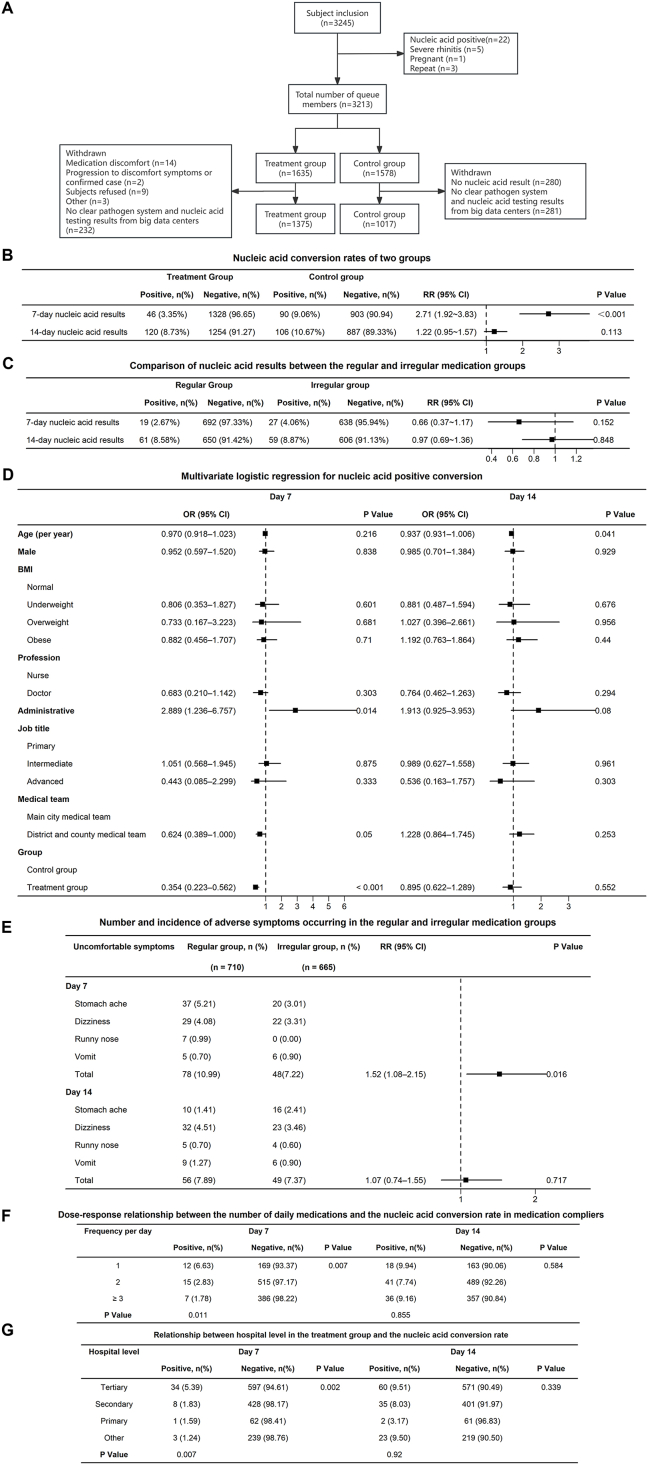
A8G6 neutralizing antibody intranasal spray offers immediate protection for healthcare workers in field hospitals.

A total of 3245 individuals were included after screening, while 31 were excluded (22 due to nucleic acid positive, 5 with severe rhinitis, 3 duplicates, and 1 pregnant individual). 2392 participants were analyzed, with 1375 in the treatment group and 1017 in the control group (Fig. 1A).

We compared the nucleic acid conversion rates between the two groups. By day 7, the treatment group (46 participants) exhibited a significantly lower nucleic acid conversion rate of 3.35% compared with the control group's (90 individuals) rate of 9.06% (*p* < 0.001). By day 14, the conversion rate in the treatment group was 8.73%, compared with 10.67% in the control group; however, this difference was not statistically significant (*p* = 0.113) (Fig. 1B).

To clarify the risk factors for nucleic acid conversion, we conducted a logistic regression analysis. At day 7, the neutralizing antibody intranasal spray significantly reduced the SARS-CoV-2 nucleic acid conversion rate (*p* < 0.001) (Fig. 1C).

A trend analysis was conducted to evaluate the relationship between average daily medication frequency and nucleic acid positive conversion rates. By day 7, variations were observed between once-daily and twice-daily dosing (*p* = 0.025), as well as between once-daily and thrice-daily dosing (*p* = 0.003), but no significant differences were observed between twice-daily and thrice-daily dosing (*p* = 0.561). However, no significant differences were observed by day 14 (Fig. 1D).

The Cochran–Armitage test was applied to assess the link between hospital level in the treatment group and nucleic acid conversion rates. Hospital level impacted conversion rates at day 7 for tertiary versus secondary hospitals (*p* = 0.005) and tertiary versus other hospitals (*p* = 0.003), but not at day 14 (Fig. 1E).

To explore the relationship between medication adherence and nucleic acid conversion rate, participants were split into regular (51.64%) and irregular (48.36%) medication groups. Baseline differences included marital status (*p* = 0.030), occupation (*p* < 0.0001), and medical team (*p* = 0.002). By days 7 and 14, there were no significant differences in nucleic acid conversion rates between the groups. By day 7, stomachache (5.21%) was prominent in regular users, while dizziness (3.01%) prevailed in irregular users. By day 14, dizziness was most common in both groups: 4.51% in regular users and 3.46% in irregular users. Significantly, only day 7 showed a difference in adverse symptom rates between the groups (*p* = 0.016) (Fig. 1F, G).

In summary, A8G6 intranasal spray provides short-term protection for high-exposure healthcare workers and is considered safe and reliable. By day 7, the treatment group exhibited a significantly lower nucleic acid conversion rate compared with that of the control group, indicating the efficacy of continuous A8G6 intranasal spray use in preventing SARS-CoV-2 infection. By day 14, the conversion rate of the treatment group was slightly lower than that of the control group, although this difference was not statistically significant. This could be attributed to difficulties in drug distribution during the pandemic, which resulted in some participants receiving only one bottle of A8G6 intranasal spray and not continuing its use after day 7. The dosage also significantly impacted the conversion rate, with once-daily users experiencing a marked decrease in efficacy compared with those using the spray twice or thrice daily. This suggests that drug concentration could notably influence the preventive effects of A8G6 intranasal spray. When hospitals were categorized by level and conversion status, tertiary hospitals exhibited significantly higher positive conversion rates compared with secondary hospitals and those with unspecified levels. This was likely due to the greater number and severity of admitted infected patients in tertiary hospitals. Interestingly, when regular usage was defined as using the intranasal spray at least once daily before and after entering isolation rooms, and deviations from this were classified as irregular usage, no significant difference in conversion rates was observed between the two groups. This implies that a certain level of protection can be achieved as long as A8G6 is used within a specific timeframe, irrespective of usage frequency. Limiting the frequency of use can reduce drug-related discomfort, even though adverse symptoms are mild and do not involve severe reactions.

In this prospective cohort study, there are some unresolved issues. Firstly, there is a significant correlation between medication dosage and outcomes; maintaining a certain concentration is essential for effectively reducing conversion rates and achieving preventive effects. However, the observations based on dosing frequency or duration revealed a relationship between drug concentration and adverse symptoms. It is necessary to find a balance between dosage and discomfort to ensure effective treatment while minimizing symptoms. Secondly, while some subgroup analyses showed differences, they did not reach statistical significance, possibly due to insufficient sample size, which may have hindered the ability to demonstrate differences between the groups. Lastly, the study sample consisted entirely of vaccinated individuals. The effectiveness of antibody injections may vary in the broader population with natural immunity. The limited sample size and its restriction to a single region and ethnicity may lead to biases in the data results.

## CRediT authorship contribution statement

**Shu Zhang:** Writing – original draft, Investigation, Data curation. **Zhigang Chu:** Project administration. **Siyuan Yang:** Writing – original draft, Data curation. **Qiuling Shi:** Project administration. **Qinghua Zhao:** Data curation. **Dawei He:** Supervision. **Hongmei Xu:** Project administration, Data curation. **Dan Zhang:** Writing – review & editing, Supervision, Resources, Methodology, Funding acquisition. **Zhu Yang:** Resources, Project administration, Funding acquisition. **Ailong Huang:** Resources, Project administration, Conceptualization.

## Funding

This study was supported by the Science and Technology Innovation Key R&D Program of Chongqing, China (No. CSTB2022TIAD-STX0013). The funders had no role in the study design; the collection, analysis, and interpretation of data; the writing of the manuscript; or the decision to publish.

## Conflict of interests

Ailong Huang is one of the Editors-in-Chief of *Genes & Diseases*, he/she has no involvement in the peer-review of this article and has no access to information regarding its peer-review. The authors declare that they have no other known competing financial interests or personal relationships that could have appeared to influence the work reported in this paper.
