# Supplementary material for: A cohort study on the A8G6 neutralizing antibody intranasal spray for preventing SARS-CoV-2 infection in healthcare workers
Source: Genes Dis. 2025 Apr 1;13(2):101617. doi: 10.1016/j.gendis.2025.101617 (PMC12594900; doi:10.1016/j.gendis.2025.101617)
Supplement: Multimedia component 1 [file mmc1.docx]

**Table 1.** Baseline characteristics of study subjects

| Characteristics | Total, n (%) (n = 2392) | Treatment group, n (%) (n = 1375) | Control group, n (%) (n = 1017) | P-value |
| --- | --- | --- | --- | --- |
| Age (mean ± SD, year) | 32.42 ± 6.62 | 32.71 ± 6.82 | 32.01 ± 6.30 | 0.010 |
| Time to complete vaccination (mean ± SD, month) | 15.52 ± 5.5 | 15.44 ± 5.62 | 15.75 ± 5.47 | 0.354 |
| Gender |  |  |  | < 0.0001 |
| Male | 555 (23.2) | 227 (16.51) | 328 (32.25) |  |
| Female | 1821 (76.13) | 1148 (83.49) | 673 (66.18) |  |
| Missing | 16 (0.67) | – | 16 (1.57) |  |
| BMI |  |  |  | 0.445 |
| Thin | 186 (7.78) | 123 (8.95) | 63 (6.19) |  |
| Normal | 1367 (57.15) | 954 (69.38) | 413 (40.61) |  |
| Overweight | 350 (14.63) | 233 (16.95) | 117 (11.5) |  |
| Obese | 71 (2.97) | 52 (3.78) | 19 (1.87) |  |
| Missing | 418 (17.47) | 13 (0.95) | 405 (39.82) |  |
| Marital status |  |  |  | 0.009 |
| Unmarried | 620 (25.92) | 404 (29.38) | 216 (21.24) |  |
| Married | 1286 (53.76) | 905 (65.82) | 381 (37.46) |  |
| Other | 78 (3.26) | 62 (4.51) | 16 (1.57) |  |
| Missing | 408 (17.06) | 4 (0.29) | 404 (39.72) |  |
| Education level |  |  |  | 0.876 |
| Technical secondary school and below | 37 (1.55) | 26 (1.89) | 11 (1.08) |  |
| Undergraduate and junior college | 1692 (70.74) | 1168 (84.95) | 524 (51.52) |  |
| Postgraduate | 240 (10.03) | 162 (11.78) | 78 (7.67) |  |
| Missing | 423 (17.67) | 19 (1.38) | 404 (39.72) |  |
| Profession |  |  |  | 0.089 |
| Nurse | 1572 (65.72) | 1019 (74.11) | 553 (54.38) |  |
| Doctor | 409 (17.1) | 253 (18.4) | 156 (15.34) |  |
| Logistics administrative staff | 133 (5.56) | 98 (7.13) | 35 (3.44) |  |
| Missing | 278 (11.62) | 5 (0.36) | 273 (26.84) |  |
| Job title |  |  |  | 0.052 |
| Primary | 1048 (43.81) | 694 (50.47) | 354 (34.81) |  |
| Intermediate | 677 (28.3) | 486 (35.35) | 191 (18.78) |  |
| Advanced | 122 (5.1) | 83 (6.04) | 39 (3.83) |  |
| Missing | 545 (22.78) | 112 (8.15) | 433 (42.58) |  |
| Medical team |  |  |  | 0.369 |
| Main city medical team | 824 (34.45) | 578 (42.04) | 246 (24.19) |  |
| District and county medical team | 1062 (44.4) | 765 (55.64) | 297 (29.2) |  |
| Missing | 506 (21.15) | 32 (2.32) | 474 (46.61) |  |
| Entry time (hours/week) | 16.60 (6.89) | 17.31 (7.12) | 16.06 (6.68) | 0.056 |
| Frequency of cabin entry (times/week) | 4.23 (1.82) | 4.38 (1.84) | 4.11 (1.79) | 0.124 |

**Table 2.** Baseline characteristics for the regular and irregular medication groups

| Characteristics | Total (n = 1375) n (%) | Regular medication group (n = 710) n (%) | Irregular medication group (n = 665) n (%) | P-value |
| --- | --- | --- | --- | --- |
| Age (years, mean (SD)) | 32.71 (6.82) | 32.90 (6.78) | 32.50 (6.87) | 0.273 |
| Time to complete vaccination (month, mean (SD)) | 15.44 (5.62) | 15.47 (5.62) | 15.42 (5.64) | 0.883 |
| Gender |  |  |  | 0.103 |
| Male | 227 (16.51) | 106 (14.93) | 121 (18.2) |  |
| Female | 1148 (83.49) | 604 (85.07) | 544 (81.8) |  |
| BMI |  |  |  | 0.340 |
| Underweight | 123 (8.95) | 68 (9.58) | 55 (8.27) |  |
| Normal | 954 (69.38) | 497 (70) | 457 (68.72) |  |
| Overweight | 233 (16.95) | 120 (16.9) | 113 (16.99) |  |
| Obese | 52 (3.78) | 21 (2.96) | 31 (4.66) |  |
| Missing | 13 (0.95) | 4 (0.56) | 9 (1.35) |  |
| Marital status |  |  |  | 0.030 |
| Unmarried | 404 (29.38) | 187 (26.34) | 217 (32.63) |  |
| Married | 905 (65.82) | 485 (68.31) | 420 (63.16) |  |
| Other | 62 (4.51) | 36 (5.06) | 26 (3.91) |  |
| Missing | 4 (0.29) | 2 (0.28) | 2 (0.3) |  |
| Education level |  |  |  | 0.128 |
| Technical secondary school and below | 26 (1.89) | 12 (1.69) | 14 (2.11) |  |
| Undergraduate and junior college | 1168 (84.95) | 621 (87.46) | 547 (82.26) |  |
| Postgraduate | 162 (11.78) | 73 (10.28) | 89 (13.38) |  |
| Missing | 19 (1.38) | 4 (0.56) | 15 (2.26) |  |
| Profession |  |  |  | < 0.001 |
| Nurse | 1019 (74.11) | 544 (76.62) | 475 (71.43) |  |
| Doctor | 253 (18.4) | 135 (19.01) | 118 (17.74) |  |
| Logistics administrative staff | 98 (7.13) | 27 (3.8) | 71 (10.68) |  |
| Missing | 5 (0.36) | 4 (0.56) | 1 (0.15) |  |
| Job title |  |  |  | 0.246 |
| Primary | 694 (50.47) | 359 (50.56) | 335 (50.38) |  |
| Intermediate | 486 (35.35) | 273 (38.45) | 213 (32.03) |  |
| Advanced | 83 (6.04) | 41 (5.77) | 42 (6.32) |  |
| Missing | 112 (8.15) | 37 (5.21) | 75 (11.28) |  |
| Medical team |  |  |  | 0.002 |
| Main city medical team | 576 (41.89) | 271 (38.17) | 305 (45.86) |  |
| District and county medical team | 767 (55.78) | 427 (60.14) | 340 (51.13) |  |
| Missing | 32 (2.33) | 12 (1.69) | 20 (3.01) |  |
| Entry time (hours/week) | 17.31 (7.12) | 17.67 (7.07) | 16.75 (7.2) | 0.379 |
| Frequency of cabin entry (times/week) | 4.38 (1.84) | 4.48 (1.84) | 4.23 (1.84) | 0.354 |
